# Microglial cytokines poison neuronal autophagy via CCR5, a druggable target

**DOI:** 10.1080/15548627.2023.2221921

**Published:** 2023-06-26

**Authors:** Beatrice Paola Festa, Farah H. Siddiqi, Maria Jimenez-Sanchez, David C. Rubinsztein

**Affiliations:** a Department of Medical Genetics, Cambridge Institute for Medical Research (CIMR), Cambridge, UK; b UK Dementia Research Institute, Cambridge Institute for Medical Research (CIMR), Cambridge, UK; c Department of Basic and Clinical Neuroscience, Maurice Wohl Clinical Neuroscience Institute, Institute of Psychiatry, Psychology and Neuroscience, King's College London, London, UK

**Keywords:** CCR5, Huntington disease, Tau, autophagy, maraviroc, microglia

## Abstract

In the prodromal phase of neurodegenerative diseases, microglia switch to an activated state resulting in increased secretion of pro-inflammatory factors. We reported that C – C chemokine ligand 3 (CCL3), C – C chemokine ligand 4 (CCL4) and C – C chemokine ligand 5 (CCL5) contained in the secretome of activated microglia inhibit neuronal autophagy via a non-cell autonomous mechanism. These chemokines bind and activate neuronal C – C chemokine receptor type 5 (CCR5), which, in turn, promotes phosphoinositide 3-kinase (PI3K) – protein kinase B (PKB, or AKT) – mammalian target of rapamycin complex 1 (mTORC1) pathway activation, which inhibits autophagy, thus causing the accumulation of aggregate-prone proteins in the cytoplasm of neurons. The levels of CCR5 and its chemokine ligands are increased in the brains of pre-manifesting Huntington disease (HD) and tauopathy mouse models. CCR5 accumulation might be due to a self-amplifying mechanism, since CCR5 is a substrate of autophagy and CCL5-CCR5-mediated autophagy inhibition impairs CCR5 degradation. Furthermore, pharmacological, or genetic inhibition of CCR5 rescues mTORC1-autophagy dysfunction and improves neurodegeneration in HD and tauopathy mouse models, suggesting that CCR5 hyperactivation is a pathogenic signal driving the progression of these diseases.

Neurodegenerative diseases, including HD and tauopathies, manifest with the accumulation of mutant and misfolded proteins in the cytoplasm of neurons, which form toxic aggregates over time. Aggregate-prone proteins causing neurodegeneration, including mutant huntingtin (mHTT) (in HD) and tau (in various dementias), are cleared via macroautophagy/autophagy. Impaired autophagy, as observed in many neurodegenerative diseases, results in defective clearance of aggregate precursors and the consequent accumulation of both soluble and aggregated species of those proteins, thereby enhancing toxicity in cells. Conversely, boosting autophagy via pharmacological or genetic interventions enhances the clearance of such aggregate-prone proteins and attenuates the deleterious phenotypes observed in animal models of these diseases. As autophagy has emerged as a promising therapeutic target for neurodegenerative diseases, there have been large efforts to dissect novel mechanisms interfering with autophagy in these conditions. Up to now, most of the studies have focused on cell-autonomous mechanisms of autophagy impairment in neurons. In the prodromal phase of neurodegenerative diseases, activated microglia secrete pro-inflammatory factors that are neurotoxic. Therefore, we asked whether these paracrine signals might negatively impact neuronal autophagy via a non-cell autonomous mechanism.

**Figure 1.**
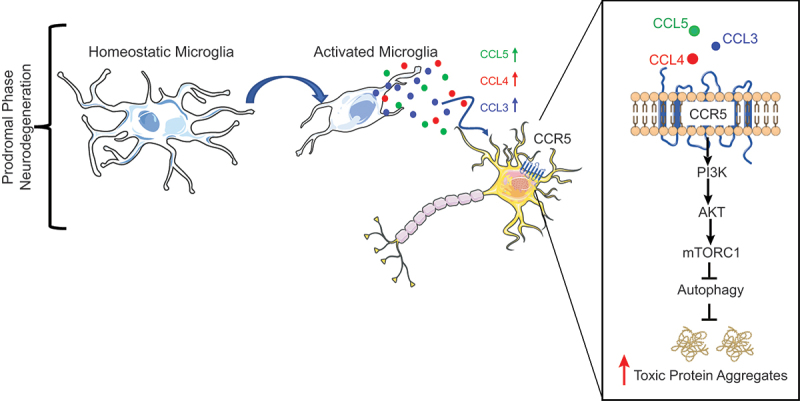
Activated microglia regulate neuronal autophagy via a non-cell autonomous mechanism. In the prodromal phase of neurodegenerative diseases, microglia switch to an activated state resulting in increased production of CCL5, CCL4 and CCL3. These chemokines bind and activate neuronal CCR5, which, in turn, inhibits autophagy by promoting PI3K-AKT-mTORC1 signaling. This enhances the buildup of toxic aggregate-prone proteins.

In cell culture studies, we found that the secretome of activated microglia inhibits neuronal autophagy [1]. Our experiments suggested that this was due to the chemokines CCL5, CCL4 and CCL3 binding to neuronal CCR5, a G-protein coupled receptor (Figure 1). This results in impaired clearance of various autophagy substrates, including the autophagy receptor sequestosome 1 (SQSTM1/p62), aggresomes, and polyQ aggregates. Mechanistically, we found that stimulation of CCR5 by its chemokines ligands triggers the PI3K-AKT-mTORC1 axis, a well-described autophagy suppressor pathway. When we blocked mTORC1 activity, we abrogated the inhibitory effect of CCR5 activation on autophagy, suggesting that mTORC1 is the predominant mediator of the CCR5 effect on this phenotype.

In the brains of pre-manifesting mouse models of tauopathy and HD, we observed microglial activation and increased levels of CCL5, CCL4 and CCL3. The neuronal levels of CCR5 were also increased in these mice. Altogether, these data suggested that the microglia-to-neuronal axis leading to CCR5 activation might be recapitulated in disease conditions, *in vivo*. In line with this hypothesis, the neuronal mTORC1 upregulation and autophagy dysregulation that we observed upon CCR5 activation in cell culture also occurred in pre-manifesting mouse models of tauopathy and HD. Genetic depletion of CCR5 in these mice rebalanced mTORC1 signaling and autophagy and prevented the accumulation of insoluble tau and mutant huntingtin (mHTT) aggregates, which are typically observed at later stages in tauopathy and HD mice. Concomitantly, we observed a significant improvement of relevant neurodegeneration-associated behavioral patterns in both mouse models.

CCR5 is a human immunodeficiency virus (HIV) co-receptor and several compounds have been developed to inhibit its function. Maraviroc, an allosteric CCR5 antagonist, is an FDA approved drug, which was previously shown to prevent CCR5 binding to chemokines. Maraviroc treatment abolished the effect of CCR5 activation by chemokines – it prevented mTORC1-autophagy dysregulation and the accumulation of aggregates both in human HeLa-CCR5-GFP cells and in mouse-derived neurons. Maraviroc rescued the mTORC1-autophagy defects observed in tauopathy and HD mouse models and markedly reduced the accumulation of sarkosyl-insoluble tau and mHTT in the brains of these animals. In addition to the rescue of histological phenotypes, maraviroc treatment also prevented the overt neuronal loss that was observed in tauopathy mice over time and significantly improved learning and memory function in all the treated animals.

Analysis of a mouse model overexpressing human mutant tau under the control of a Tet-Off system, revealed that pathogenic tau expression directly correlates with CCR5 levels and inversely correlates with the levels of autophagy. This prompted us to investigate the hypothesis that CCR5 might be itself a target of autophagy. We found that both inhibition of lysosomal degradation via Bafilomycin A1 and blocking of autophagosome biogenesis using the Unc−51 like autophagy activating kinase (ULK1/2) inhibitor, SBI−0206965, increased CCR5 levels in neurons. Conversely, by enhancing autophagy via physiological stimuli, such as serum or glucose starvation, we promoted CCR5 degradation. We also showed that CCR5 levels were abnormally increased in cells depleted of key autophagy proteins such as autophagy-related protein 16–1 (ATG16L1) and autophagy-related protein 5 (ATG5) and re-introducing the expression of these autophagy-related proteins restored normal CCR5 levels. Altogether, these data suggest that CCR5 is a substrate of autophagy.

CCR5 is degraded in a non-canonical manner via autophagy. Typically, autophagy substrates that are free in the cytoplasm are engulfed by autophagosomes. CCR5, in a manner similar to what we previously described for the transferrin receptor, is a transmembrane protein that is endocytosed from the plasma membrane and enters the autophagy pathway after trafficking to the recycling endosomes where autophagosome formation occurs. CCR5 activation by its cognate ligands does not impact on the receptor trafficking but impairs autophagosome formation at the recycling endosomes, thereby blocking CCR5 degradation, which causes its accumulation. In other words, CCR5 activation by chemokines triggers a positive feed-back loop that might result in an abnormal amplification of CCR5 signaling, with potential harmful effects for neuronal health. We suggest that this detrimental loop might be activated in the pre-manifesting stage in neurodegenerative disease mouse models, when CCR5 is chronically exposed to high concentrations of CCL5, CCL4 and/or CCL3.

Our results reveal a new role for activated microglia in the non-cell autonomous downregulation of neuronal autophagy [1]. This pathogenic microglia-neuron crosstalk is prominent in the prodromal phase of neurodegeneration and is potentially self-amplifying but may be suitable for therapeutic approaches.

## Data Availability

This is a Punctum summarizing data described in the referenced paper https://doi.org/10.1016/j.neuron.2023.04.006.

## Abbreviations

AKT or PKB: Protein kinase B
ATG5: Autophagy-related protein 5
ATG16L1: Autophagy-related protein 16–1
CCL3: C – C chemokine ligand 3
CCL4: C – C chemokine ligand 4
CCL5: C – C chemokine ligand 5
CCR5: C – C chemokine receptor type 5
HIV: Human immunodeficiency virus
mTORC1: Mammalian target of rapamycin complex 1
PI3K: Phosphoinositide 3-kinase
SQSTM1/p62: Sequestosome 1
ULK1/2: Unc−51 like autophagy activating kinase
